# A Protest against Extracting Pulpless Teeth

**Published:** 1915-05

**Authors:** C. N. Johnson


### A PROTEST AGAINST EXTRACTING PULPLESS TEETH.

BY C. N. JOHNSON, L.D.S., D.D.S.

Time and again has the fact been emphasized that bad mouth conditions were a menace to the patient, that foci of infection in the gums or sockets of the teeth were likely to spread infection elsewhere. In season and out of season has the dentist claimed that the teeth were of greater importance than the physician thought they were. Always and everywhere has he preached mouth hygiene till he has been termed a fanatic about it. Now has come "the winter of our discontent," and certain dentists and certain physicians have suddenly turned their energies and are clambering over the bounds of reason and sanity in their mad rush to put their finger on a pulpless tooth and condemn it.

The simple facts are these: That while grave injury has undoubtedly been done by the injudicious retention of badly filled and diseased pulpless teeth, and while the profession is open to criticism for the imperfect manner in which many pulpless teeth have been treated, there is no question whatever that most pulpless teeth are amenable to proper treatment and can be made useful, comfortable and safe for the patient, to his very great advantage. Can we not have reform in this matter without fanaticism? Can we not consecrate ourselves to a calm and judicious study of the problem which confronts us so that we may sift the chaff from the wheat and preserve the wheat? Must we throw reason to the winds and run amuck among our patients, sowing havoc wherever we go? No one questions the sincerity or honesty of purpose of the men who are doing this thing. Their intentions are undoubtedly good, but we must not forget that there is a certain very undesirable place which is said to be paved with good intentions.

What is to become of the people from whose mouths useful teeth are being taken? Are they to have bridge-work on teeth with pulps alive or are they to be condemned to artificial dentures all their days? Either alternative can not be alluring to the thoughtful man. If this panic of extraction is to continue, God help the people.—Extract from editorial in May *Review*.

